# Engineering brain organoids for functional validation and translational applications: Construction strategies, vascularization, and standardization

**DOI:** 10.1002/btm2.70164

**Published:** 2026-08-19

**Authors:** Guohong Huang, Chenfei Lu, Zihan Jin, Yunchuanxiang Huang, Xinya Du, Xixi Hu, Hui Peng, Shanrun Wang, Lin Wen, Juhui Qiu, Guixue Wang, Chuanrong Zhao

**Affiliations:** ^1^ Key Laboratory for Biorheological Science and Technology of the Ministry of Education, National Local Joint Engineering Laboratory for Vascular Implants, Bioengineering College of Chongqing University Chongqing China; ^2^ Institute of Panvascular Biology, JinFeng Laboratory Chongqing China

**Keywords:** blood–brain barrier, brain organoids, disease modeling, functional validation, standardization, translational bioengineering, vascularization

## Abstract

Brain organoids provide three‐dimensional human cellular systems that can reproduce selected features of early neural development, regional patterning, cellular diversification, and emerging neural activity more effectively than conventional two‐dimensional cultures. However, their translational value depends not only on morphological resemblance to brain tissue, but also on whether construction strategies, functional validation, reproducibility, and application‐specific model fitness are appropriately aligned. This structured narrative review synthesizes representative engineering strategies for brain organoid construction and examines how cell source, embryoid body formation, extracellular matrix support, patterning strategy, culture platform, vascularization, and cellular complexity influence functional validation and translational applicability. We further organize functional assessment into a hierarchical validation framework that includes morphology and growth, lineage and regional identity, tissue viability, synaptic maturation, electrophysiological activity, neurochemical signaling, BBB‐like function, and omics‐based benchmarking. These advances support the use of brain organoids in developmental biology, neurological disease modeling, drug screening, neurovascular research, and exploratory biohybrid interfaces, although their interpretation remains constrained by immature cellular states, incomplete vascular perfusion, batch variability, and limited standardization. Overall, this review reframes brain organoids as engineered biological platforms whose value should be judged by the alignment among construction strategy, biological benchmark, functional readout, and intended translational application. The emphasis is comparative conceptual synthesis of engineering strategies and multi‐layer functional validation rather than systematic quantitative meta‐analysis.

**Translational Impact Statement**

This review establishes an engineering‐to‐function perspective for evaluating brain organoid platforms by linking controllable construction variables with the functional evidence required for specific biomedical applications. By integrating considerations of disease modeling, CNS drug screening, neurovascular and BBB‐like research, and emerging biohybrid systems, this review highlights the importance of application‐specific validation, reproducibility, and standardization. This framework provides practical guidance for selecting and optimizing brain organoid platforms with improved translational reliability.

## 1 INTRODUCTION

Brain organoids are three‐dimensional human cellular systems generated from pluripotent stem cells through self‐organization and guided or unguided neural differentiation.^1^ Compared with conventional two‐dimensional cultures, brain organoids better reproduce selected features of early neural development, including neuroepithelial organization, regional patterning, neural progenitor expansion, neuronal differentiation, and emerging network activity.^2^ These properties make brain organoids useful platforms for studying human neurodevelopment, neurological disease mechanisms, drug responses, and cell–cell interactions that are difficult to model in conventional monolayer cultures.^3^ However, current brain organoids remain simplified and immature models, and their biological relevance depends strongly on construction strategy, culture conditions, functional validation, and reproducibility.

Different construction strategies can generate brain organoids with distinct biological properties.^4^ Cell source, embryoid body formation, extracellular matrix support, patterning strategy, culture platform, vascularization, and incorporation of additional cell types all influence organoid regional identity, cellular composition, maturation state, viability, and functional activity.^5^ Therefore, morphological resemblance to brain tissue is not sufficient to define a useful brain organoid model. A reliable model should be evaluated according to whether its construction strategy matches the intended biological question and whether its key properties are supported by appropriate functional readouts.^6^

Existing reviews have extensively summarized brain organoid protocols, disease models, and emerging applications.^7^ However, a central issue remains insufficiently integrated: how engineering variables in organoid construction determine downstream functional validation, reproducibility, and translational reliability.^8^ This issue is particularly important for vascularized organoids, BBB‐like models, electrophysiological assessment, drug screening, and exploratory organoid–computer interfaces, where strong functional claims require multi‐level evidence rather than morphology or marker expression alone.

Most recent reviews concentrate on developmental protocols, disease phenotypes, or a single enabling technology.^7^, ^9^, ^10^, ^11^ Fewer studies bring engineering variables, functional evidence, vascular or BBB features, and reproducibility into the same discussion. This review addresses that gap by asking a practical question throughout: what evidence is needed to support the claim made for a particular organoid design?

Accordingly, this review proposes an engineering‐to‐function validation framework, in which controllable construction variables are evaluated according to their effects on biological benchmarks, measurable functional outputs, and application‐specific translational requirements.

This review focuses on the relationship between brain organoid construction and functional evaluation, with particular attention to how different engineering strategies shape regional identity, cellular composition, maturation status, and translational potential. Rather than simply cataloging published studies, we aim to connect organoid‐generation strategies with the functional readouts required to support specific biological and biomedical claims. We therefore summarize key variables, including cell source, patterning strategy, culture platform, vascularization, and cellular complexity, and discuss how these factors influence morphological, molecular, electrophysiological, BBB‐like, and omics‐based validation.^6^ Through this perspective, brain organoids are considered not merely as three‐dimensional structures resembling brain tissue, but as engineered biological platforms that should be designed, validated, and interpreted according to the intended research or translational application.^12^

## 2 REVIEW SCOPE AND LITERATURE SELECTION

This article is a structured narrative review rather than a systematic review or quantitative meta‐analysis. Relevant literature was identified through targeted searches of PubMed/MEDLINE, Web of Science Core Collection, Scopus, and Embase from database inception to 30 June 2026. Search terms combined brain organoid, cerebral organoid, cortical organoid, neural organoid, and assembloid with engineering, extracellular matrix, bioreactor, microfluidics, perfusion, patterning, vascularization, blood–brain barrier, functional validation, electrophysiology, reproducibility, drug screening, and biohybrid computing. Studies were included when they directly informed human brain‐organoid construction, validation, application, or standardization; non‐neural organoids and two‐dimensional neural cultures were used only as relevant comparators. Additional references were identified through citation tracking. Given the heterogeneity of the evidence, studies were interpreted according to whether the reported methods and functional readouts supported the claims made, rather than through formal risk‐of‐bias scoring.

## 3 BIOLOGICAL BENCHMARKS AND STRUCTURAL FEATURES OF BRAIN ORGANOIDS

Brain organoid construction should begin with a clear definition of the biological features that the model is expected to reproduce. Regional identity, cellular composition, developmental trajectory, neural network activity, and neurovascular or BBB‐related organization are useful benchmarks, but each should be interpreted as a partial and application‐specific feature rather than a complete reconstruction of the human brain. The brain is a highly complex, multiscale system characterized by interconnected and functionally specialized regions, a highly heterogeneous cellular population, and a sophisticated regulatory architecture comprising neural and vascular networks.

### 3.1 Regional identity and structural heterogeneity

The human brain is a complex system composed of multiple brain regions that exhibit high anatomical and functional segregation. Structures such as the cerebral cortex, basal ganglia, thalamus, hippocampus, hypothalamus, midbrain, and cerebellum show significant differences in their cellular composition, gene expression profiles, local microcircuits, and cognitive and behavioral functions. Furthermore, these regions can be subdivided into functionally specialized subregions, demonstrating clear spatial partitioning, regional diversity, and heterogeneity.^13^ At the same time, these structurally and functionally distinct brain regions are integrated into multiple, overlapping large‐scale functional networks through dense short‐ and long‐range axonal projections. This allows the brain to maintain a dynamic balance between regional specialization and global integration, supporting multiregional collaborative functions such as sensory processing, motor control, emotional regulation, and higher‐order cognition.^14^ Therefore, multiregional and multiscale structural and functional heterogeneity is a core feature of the complexity of the brain and a critical dimension that requires special attention when modeling the brain in vitro with brain organoids.

Brain organoids have been used to model selected cellular and architectural features of specific developing brain regions, providing an in vitro system for investigating certain aspects of early human brain development. As early as 2013, Lancaster et al. generated cerebral organoids from human pluripotent stem cells through neuroectodermal differentiation, producing three‐dimensional tissues with features reminiscent of early cortical organization.^2^ Embryonic stem cells (ESCs) are typically neutrally inducible in vitro through dual SMAD pathway inhibition, simultaneously inhibiting both the bone morphogenetic protein (BMP) and transforming growth factor‐β (TGF‐β) signaling pathways to efficiently differentiate into neural progenitor cells (NPCs).^15^ Subsequently, signaling molecules such as fibroblast growth factor (FGF), retinoic acid (RA), WNT, and sonic hedgehog (SHH) promote region‐specific lineage specification. Ultimately, the resulting neural aggregates are embedded in a three‐dimensional extracellular matrix, such as Matrigel, and maintained under rotary or bioreactor‐based culture conditions to improve medium mixing and oxygen and nutrient delivery, thereby supporting tissue expansion and further maturation of brain organoids,^2^, ^16^, ^17^ which support selected structural and functional features of the developing human brain. The methods used to generate brain organoids from pluripotent stem cells include the use of multiple neuronal cell populations, such as neural stem cells and astrocytes, which form basic neural structures.^18^ These cell populations are not confined to a single outer layer and should not be interpreted as forming an anatomical equivalent of cortical layer 1; instead, their spatial distribution varies with organoid type, developmental stage, and regional patterning.^18^, ^19^ Excitatory cortical‐like neurons are among the neuronal populations generated in many cerebral and cortical organoid protocols, but their subtype composition, spatial distribution, and maturation vary among models. These neurons align in a cortical‐like arrangement, forming functional regions that resemble those in the brain cortex. Rather than constituting a discrete inner layer or a core region equivalent to the cerebral cortex, early cerebral organoids commonly contain ventricular‐like lumens surrounded by NESTIN‐, SOX2‐, and PAX6‐positive neural progenitor and neuroepithelial cells, from which differentiating neurons migrate toward more peripheral cortical plate‐like zones.^18^, ^19^

### 3.2 Cell composition

**FIGURE 1:**
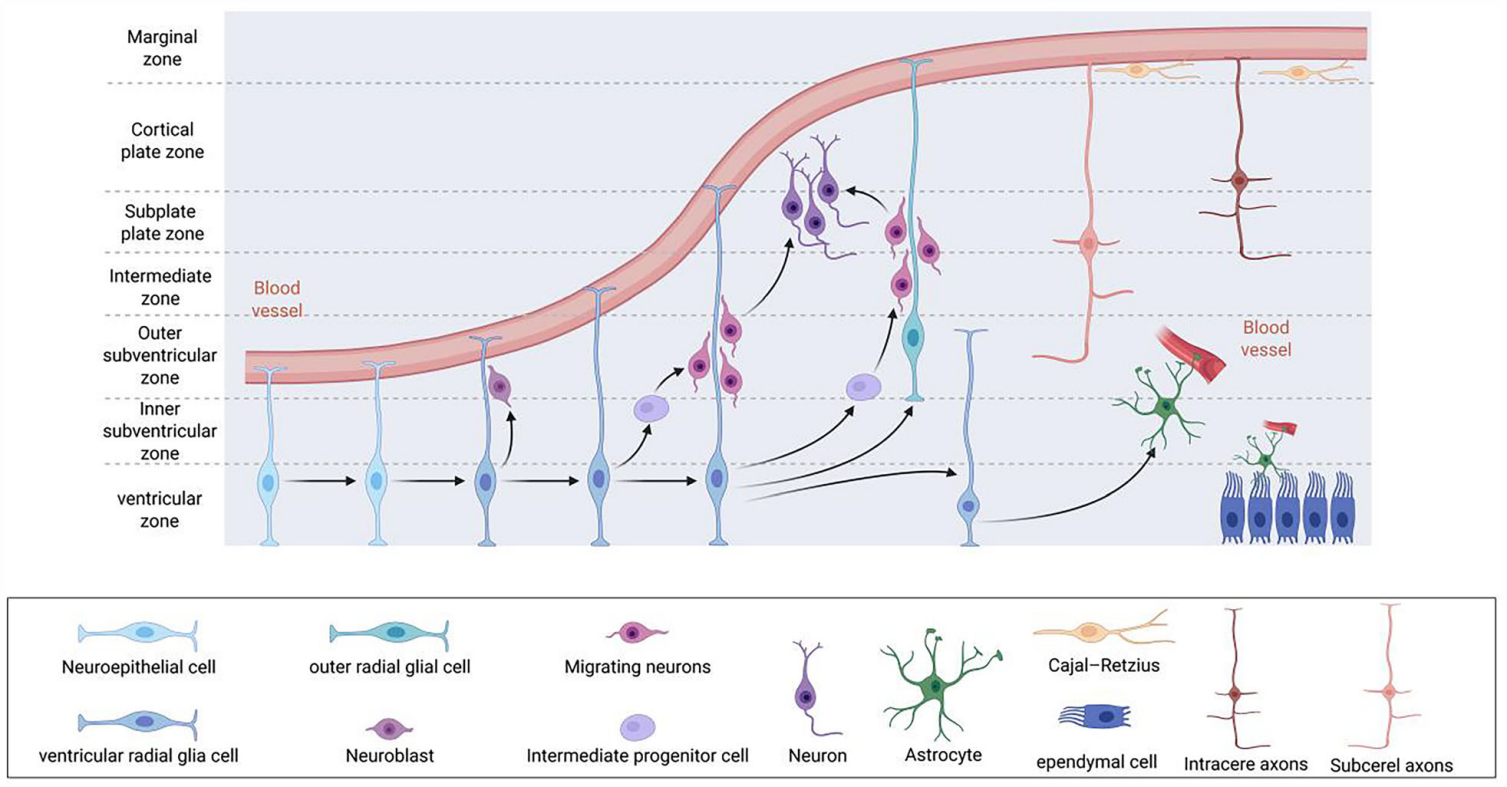
Neuroepithelial cells (NPCs) undergo symmetric cell division to generate an initial pool of cortical progenitor cells, which subsequently transform into ventricular radial glial cells (vRGCs). The vRGCs then begin asymmetric divisions, producing one new vRGC and one newborn neuroblast. The earliest generated neurons migrate to form the preplate. The neurons generated later split the preplate into the marginal zone (MZ) and the subplate (SP). As neurogenesis progresses, RGCs give rise to multiple neuronal subtypes through successive asymmetric divisions. In addition, some daughter cells of RGCs differentiate into intermediate progenitor cells (IPCs) or outer radial glial cells (oRGCs) in the subventricular zone (SVZ). Moreover, at the end of neurogenesis, the radial scaffold is dismantled, and the NPs enter the gliogenic phase, generating astrocytes in the cortex and the SVZ and giving rise to a layer of ependymal cells (ependymal layer, EL).^19^ This figure was constructed using BioRender.

The ventricular zone (VZ) is composed mainly of neuroepithelial cells and their derived radial glial cells and is considered the primary neural stem cell population in the embryonic cortex.^20^ Adjacent to the VZ, the subventricular zone (SVZ) is composed mainly of intermediate progenitor cells, which no longer directly contact the ventricles and represent the most abundant transitional progenitor cell type during cortical neurogenesis.^21^ In humans and other primates, the lateral part of the SVZ further differentiates into a distinct outer subventricular zone (OSVZ), which is enriched with outer radial glial cells (oRGs) and constitutes a significant progenitor cell population in the human cortex.^22^ Moving outwards, the intermediate zone (IZ) mainly consists of immature neurons in a migratory state and some axonal structures, reflecting that this area primarily contains cells involved in migration and connectivity formation.^19^ The outermost cortical plate (CP) is composed of terminally differentiated excitatory cortical neurons, containing projection neuron populations of different layers.^23^ In the marginal zone (MZ) at the surface of the cortex,^24^ Cajal–Retzius cells are mainly distributed and are characterized by Reelin, a unique neuronal population present in the early cortex (Figure 1).^25^

### 3.3 Neural network formation

In brain organoids, network formation is commonly assessed using synaptic markers, spontaneous calcium transients, extracellular field potentials, and coordinated bursting activity. Previous studies have reported that cortical organoids can develop spontaneous electrical activity and network‐level oscillatory patterns during long‐term culture, suggesting that these models may capture certain features of early human cortical network development.^26^ However, these activities should be interpreted as immature and simplified network properties rather than equivalents of fully developed brain circuits.

Abnormal network activity is associated with neurological disorders characterized by altered neuronal excitability. Epilepsy, for example, is related to recurrent seizure activity arising from neuronal hyperexcitability and hypersynchrony. Brain organoid models derived from patients with neurodevelopmental disorders have been used to investigate epileptiform‐like activity and altered network dynamics, particularly when combined with calcium imaging or multielectrode array recordings.^27^ These models provide a useful platform for studying disease‐related changes in neuronal excitability and circuit organization, although their interpretation should take into account organoid type, maturation stage, and the lack of long‐range anatomical inputs.

### 3.4 Blood–brain barrier and neurovascular benchmarks

**FIGURE 2:**
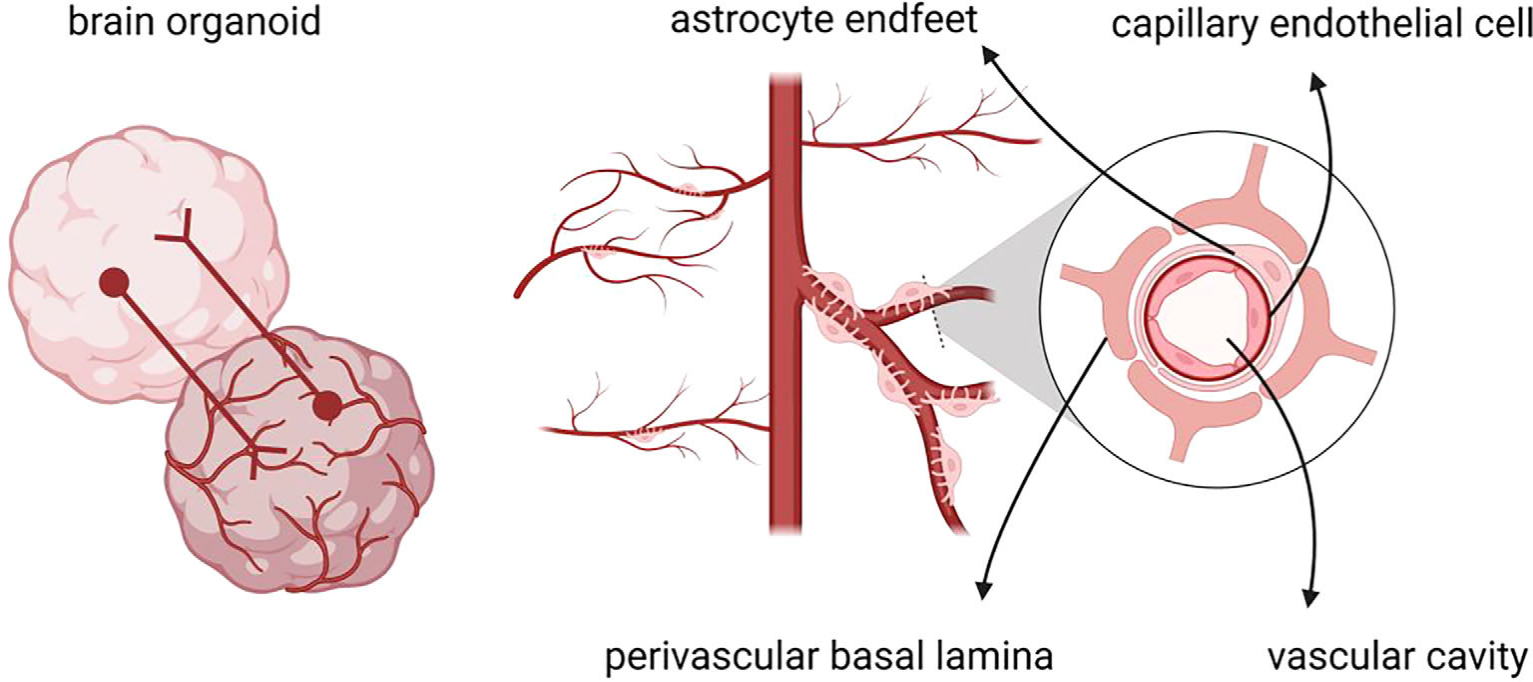
Schematic of the blood–brain barrier and neurovascular network. This figure was constructed using BioRender.

The blood–brain barrier (BBB) is an important functional feature of the cerebral microvasculature and contributes to central nervous system homeostasis by regulating molecular and cellular exchange between the bloodstream and neural tissue.^28^ The BBB is mainly formed by brain microvascular endothelial cells and is regulated by pericytes, astrocytic endfeet, basement membrane components, and neuronal and glial signals within the neurovascular unit (NVU) (Figure 2).^29^

BBB development is closely associated with vascular ingrowth, endothelial specialization, pericyte recruitment, and later astrocyte‐related maturation. Early brain vascular formation is influenced by neuroepithelial cells and neural progenitor populations, among which radial glial cells have been implicated in nascent vessel stabilization and cortical vascular organization.^30^ Therefore, VEGF signaling is more appropriately discussed in the context of developmental angiogenesis and vascular remodeling rather than being mainly attributed to astrocytes during early brain development. Wnt/β‐catenin signaling is associated with endothelial barrier specification,^31^ tight‐junction formation, and suppression of transcytosis^31^; PDGF‐B/PDGFRβ signaling contributes to pericyte recruitment and vascular stabilization^32^; and Sonic Hedgehog signaling has been linked to BBB integrity and neurovascular immune homeostasis.^33^

Conventional brain organoids lack a perfusable vascular network. Endothelial‐cell incorporation, ETV2‐mediated programming, vascular‐organoid fusion, and transplantation can introduce vascular or BBB‐like components,^8^ but these models differ substantially in vessel maturity and perfusion. Vascularization should therefore be distinguished from functional BBB formation; the engineering approaches are compared in Section 5.

BBB‐related brain organoid models may be useful for studying neurovascular development, barrier maturation, neuroinflammation, ischemic injury, and drug delivery across the BBB. Their functional evaluation usually requires a combination of endothelial markers, tight‐junction proteins, transporter expression, permeability assays, and transendothelial electrical resistance measurements. However, current vascularized or BBB‐like brain organoids remain simplified models and may not fully capture the cellular composition, perfusion dynamics, or regional heterogeneity of the human BBB.

## 4 CONSTRUCTION STRATEGIES FOR BRAIN ORGANOIDS

Brain organoid construction should be organized according to distinct engineering variables rather than a single mixed classification. These variables include biological inputs (cell source and genetic background), microenvironmental factors (ECM and matrix support), engineering platforms (dynamic culture and microfluidic systems), patterning and assembly strategies, and vascular or BBB‐like engineering approaches. Accordingly, the following subsections separate biological raw materials and matrix support, dynamic culture equipment, and patterning/assembly complexity; vascular and BBB‐like engineering is considered independently in Section 5.

### 4.1 Biological inputs and microenvironmental support

Brain organoids are self‐organizing three‐dimensional structures generated from pluripotent stem cells with cellular and tissue architectures that resemble those of the embryonic human brain.^7^ Undifferentiated human embryonic stem cells (hESCs) and human induced pluripotent stem cells (hiPSCs) possess robust self‐renewal capacity and can differentiate into specific cell types.^34^ hESCs are derived from the inner cell mass of blastocyst‐stage embryos and are often used to generate “healthy control” organoids for studying normal human organogenesis and basic pharmacological effects. hiPSCs are derived by reprogramming somatic cells to a pluripotent state through the overexpression of a key set of transcription factors. Because hiPSCs can be easily generated by reprogramming somatic cells from different patients, retaining individual‐specific genotypes and readily combining with CRISPR/Cas9 technology for genetic editing, hiPSC‐derived organoids have become a major platform for disease modeling and precision drug response prediction. Additionally, pluripotent stem cell‐derived organoids are suited to modeling early developmental processes that are otherwise difficult to investigate directly.^35^, ^36^ hiPSCs are typically maintained in two‐dimensional culture under either feeder‐dependent or feeder‐free conditions, and these conditions influence both the course of differentiation and developmental potential.^37^ Under feeder‐free conditions, microenvironmental cues (e.g., medium composition) can be tuned to bias lineage commitment toward the neural fate.^15^, ^38^ HiPSCs are subsequently suspended in culture and encouraged to aggregate in standard U‐ or V‐bottom well plates to form embryoid bodies (EBs).^39^ EBs arise via self‐aggregation or can be produced via microfluidic approaches.^40^ As early multicellular aggregates with neuroectodermal potential, EBs are often embedded in an extracellular matrix (ECM), such as Matrigel, to provide structural support, promote morphogenesis and tissue growth, and facilitate further organoid development.^9^ Differentiation of neural progenitors occurs via unguided or guided protocols. In unguided protocols, EBs rely on intrinsic cues to self‐organize into multiple brain regions (e.g., the cerebral cortex, telencephalon, and choroid plexus), generally yielding heterogeneous and less predictable patterns. Guided protocols add defined morphogens and modulate key signaling pathways (e.g., Wnt and Sonic Hedgehog^38^) on a controlled schedule to direct regional specification toward selected brain areas, such as the cortex or telencephalon. Following successful induction of neural progenitors, organoid cytoarchitecture progressively emerges and can include neuronal as well as nonneuronal cell types.^6^, ^41^

Matrigel remains widely used because it supports neuroepithelial expansion and three‐dimensional morphogenesis.^16^ However, its undefined composition and batch‐to‐batch variability limit reproducibility and complicate translational standardization. Defined hydrogels with tunable stiffness, degradability, and ligand composition may provide more controllable alternatives for future brain organoid engineering.^42^

### 4.2 Dynamic culture platforms

Brain organoids are three‐dimensional self‐organizing structures that require in vitro culture. However, during long‐term culture, different platforms are prone to necrosis and hypoxia, which limits organoid growth and viability. To generate complex organoids and model related human diseases, the culture system should ensure efficient delivery and diffusion of oxygen and nutrients and provide a biomimetic microenvironment to recapitulate human brain organogenesis in vitro.^43^

#### 4.2.1 Bioreactor culture

**FIGURE 3:**
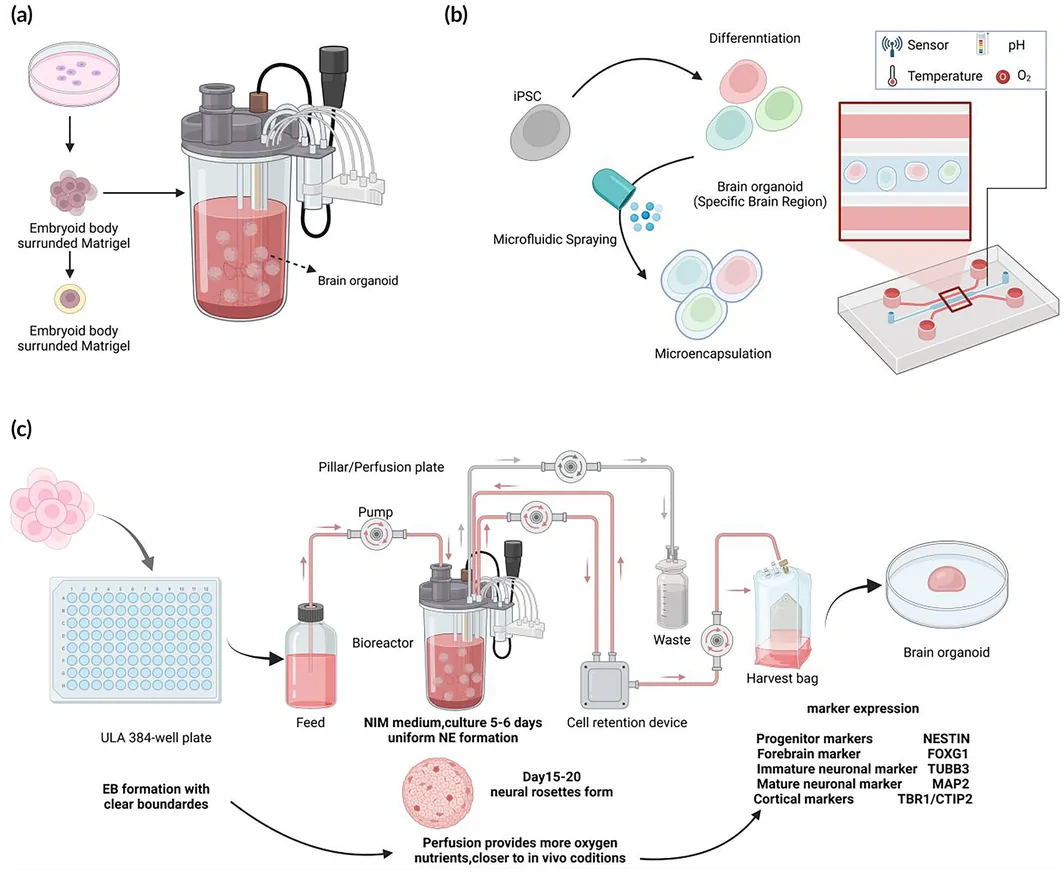
Representative culture platforms for brain organoid generation and maturation. (a) Suspension or bioreactor‐based culture for improving medium mixing and mass transfer. (b) Microfluidic‐assisted culture and organoid‐on‐chip platform for controlled fluid flow, compartmentalization, and environmental regulation. (c) Pillar/perfusion plate‐based dynamic culture for improving medium exchange, oxygenation, and organoid handling. This figure was constructed using BioRender.

Human brain organoids exhibit highly complex architectures with region‐specific and cortical‐like structures, enabling in vitro modeling of developmental processes and disease mechanisms. Early protocols aggregated pluripotent stem cells under defined conditions to form embryoid bodies (EBs), embedded the EBs in Matrigel, and transferred the Matrigel‐encapsulated EBs to dishes placed on a spinning/rotating bioreactor for suspension culture. Orbital motion promotes homogeneous mixing of the medium, supports normal EB dynamics, and ensures adequate exposure to oxygen and nutrients. Through suspension culture, EBs progressively develop into brain organoids (Figure 3a).^2^, ^16^, ^17^ Although dish‐based suspension culture can generate brain organoids, this approach provides limited control over aggregate size, oxygen and nutrient gradients, and medium exchange.^43^ These limitations may contribute to inter‐organoid variability, central hypoxia or necrosis during long‐term culture, and relatively prolonged maturation timelines before functional readouts can be obtained.

Collectively, bioreactor‐based culture is useful for improving mass transfer and scalability, but its benefit should be validated by quantitative readouts such as organoid size distribution, hypoxia, necrosis, viability, lineage composition, and electrophysiological maturation.^4^, ^17^

#### 4.2.2 Microfluidic culture

Microfluidics manipulates fluids via miniaturized devices, which include chip‐scale systems composed of microchannels, microvalves, and sensors. Brain organoids cultured in such devices, often termed organoids‐on‐a‐chip, provide controlled platforms for regulating flow, compartmentalization, nutrient delivery, drug exposure, and on‐chip sensing.^44^ By controlling channel geometry and valve operations, microfluidic platforms regulate flow rates and reaction conditions. Culturing organoids within these devices creates a favorable growth milieu in which critical conditions, such as temperature, pH, nutrient supply, oxygen delivery, and efficient waste removal, can be controlled or monitored, thereby supporting cellular and tissue growth and function.^45^

Microfluidic culture is often combined with hydrogels that provide ECM‐like structural and biochemical support.^45^, ^46^ Controlled flow can improve nutrient delivery, waste removal, compartmentalization, and drug exposure, while microcapsule or microwell formats can help organize developing tissues (Figure 3b).^47^ These advantages are offset by device‐to‐device variation, bubble formation, molecular adsorption, limited throughput, and hydrogel variability, all of which should be considered when microfluidic systems are compared with suspension culture.

However, the application of microfluidic systems in brain organoid research remains relatively early compared with organ‐on‐a‐chip models, and further studies are needed to evaluate their reproducibility, scalability, and effects on regional specification and functional maturation.

#### 4.2.3 Dynamic culture of pillar/perfusion plates

Pillar plates provide a parallel format for EB retention, neural induction, expansion, and maturation.^48^ After formation in ultralow‐attachment plates, EBs are transferred to pillar arrays and exposed to sequential neural media. Evidence from a dynamic microfluidic cerebral‐organoid platform indicates that continuous flow can improve modeled oxygen supply and reduce necrotic regions compared with static well‐plate or dish culture.^49^ The plate format simplifies handling and supports higher‐throughput culture (Figure 3c). Reported organoids form neuroepithelial structures and progress toward neuronal differentiation, but retention efficiency, differentiation timing, platform‐specific gradients, and between‐well reproducibility still require systematic evaluation.

### 4.3 Patterning strategies and multicellular assembly approaches

**FIGURE 4:**
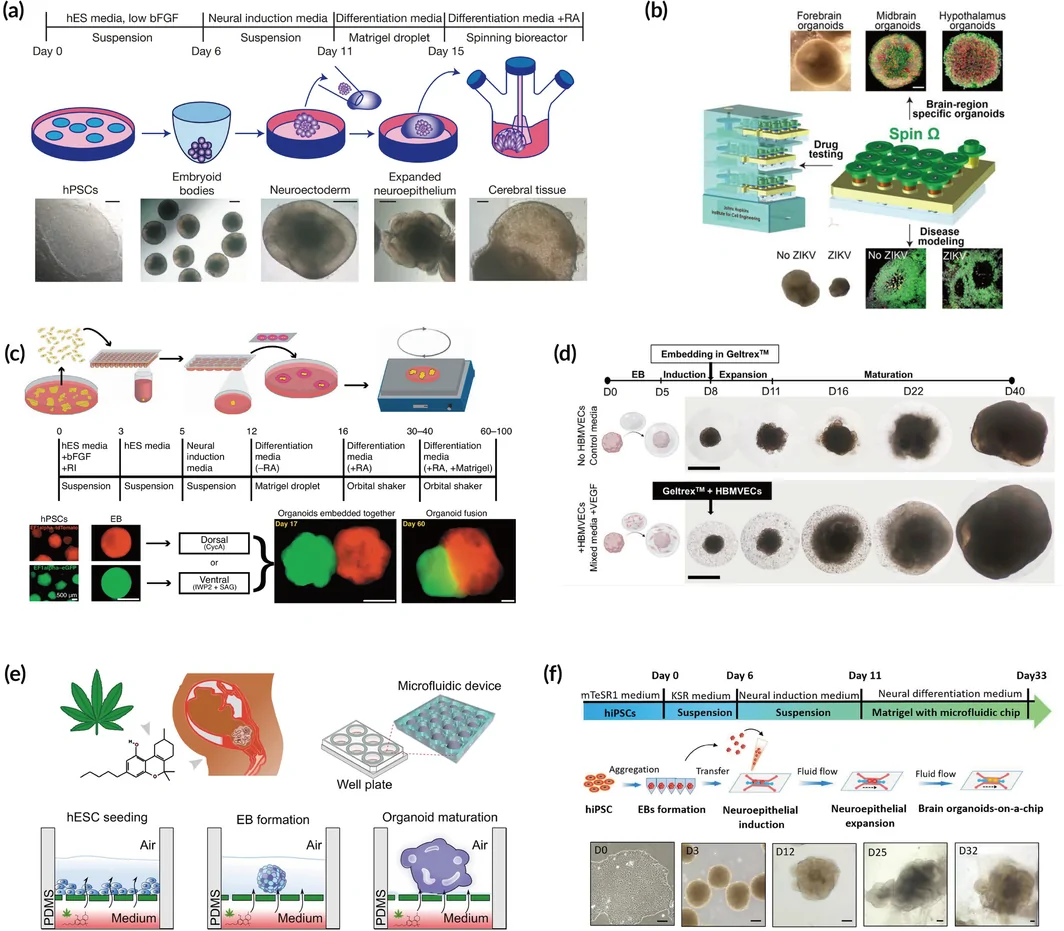
Representative strategies for increasing structural and cellular complexity in brain organoid culture. (a) Generation of unguided cerebral organoids from human pluripotent stem cells through embryoid body formation, neural induction, three‐dimensional matrix embedding, and rotating culture.^2^ (b) Generation of brain region‐specific organoids using defined patterning cues and dynamic three‐dimensional culture conditions.^4^ (c,d) Assembly of region‐specific spheroids or organoids to generate assembloid‐based models for studying interregional interactions and cell migration.^3^ (e) Microfluidic‐assisted assembly and culture of human brain organoids for modeling prenatal cannabis exposure.^50^ (f) Schematic workflow and representative microscopy images of hiPSC‐derived brain organoid‐on‐chip development for modeling prenatal nicotine exposure.^51^ Figure adapted from multiple sources under Creative Commons Attribution License.

Brain organoid models can be discussed from the perspective of structural and cellular complexity according to their construction strategies, including unguided cerebral organoids, region‐specific organoids, assembloid‐based systems, and microfluidic‐assisted assembly platforms (Figure 4a–f). This classification emphasizes how regional identity, cellular composition, spatial organization, and intercellular interactions are introduced into organoid models, rather than defining them by the number of cell types included.

Unguided cerebral organoids are generally generated from human pluripotent stem cells through embryoid body formation, neural induction, extracellular matrix embedding, and three‐dimensional culture (Figure 4a).^52^ These systems largely depend on intrinsic developmental programs and may generate heterogeneous neural tissues containing features of several developing brain regions, including cortical, telencephalic, and choroid plexus‐like identities.^53^ Because regional patterning is not strictly predetermined, unguided cerebral organoids are useful for examining selected aspects of early neurodevelopment, although their cellular composition and regional identity may vary among organoids and experimental batches.

Region‐specific organoids are produced by applying defined patterning cues to guide human pluripotent stem cells toward selected neural identities(Figure 4b).^41^ Modulation of developmental signaling pathways, including WNT, SHH, FGF, BMP, and retinoic acid‐related pathways, can bias organoid differentiation toward cortical, ventral forebrain, midbrain, hypothalamic, or choroid plexus‐like lineages.^53^ Compared with unguided cerebral organoids, region‐specific models provide a more controlled setting for investigating regional specification, neuronal differentiation, and disease‐associated phenotypes within defined developmental contexts.

Assembloid‐based systems are generated by combining regionally specified organoids or spheroids to study selected interregional and cell–cell interactions (Figure 4c,d).^3^ For example, dorsal forebrain or cortical spheroids have been assembled with ventral forebrain or subpallial spheroids to examine interneuron migration and excitatory–inhibitory circuit interactions.^3^ Other approaches incorporate non‐neuronal components, such as microglia, endothelial cells, pericyte‐like cells, or vascular‐like structures, to model aspects of neuroimmune or neurovascular interactions.^8^, ^54^, ^55^, ^56^ These systems increase the biological complexity of brain organoid models, but they remain simplified in vitro representations of the developing human brain.

Microfluidic‐assisted assembly platforms provide another engineering strategy for organizing organoid‐forming tissues or regionally specified organoids within defined spatial architectures (Figure 4e,f).^47^, ^50^, ^51^ In these systems, microfluidic devices can be used to position organoids in microwell or micropillar‐based structures, support controlled tissue assembly, and maintain on‐chip culture under more defined fluidic conditions. Such platforms may help study spatial organization, tissue fusion, and interregional interactions in a more controllable microenvironment, although their effects on regional specification, long‐term maturation, and functional integration require further validation.

During brain organoid development, neural lineage populations, including neural stem cells, radial glial cells, intermediate progenitor cells, neurons, astrocytes, and oligodendrocyte lineage cells, may emerge in a temporally organized manner that resembles certain aspects of embryonic brain development.^57^ In cortical organoid systems, deep‐layer projection neuron markers often appear before upper‐layer neuronal markers, whereas glial populations, including astrocytes and oligodendrocyte lineage cells, usually arise at later stages.^57^ These developmental sequences provide useful criteria for evaluating organoid maturation, but they should be interpreted as partial developmental features rather than a complete reconstruction of brain architecture or function.

## 5 VASCULARIZATION AND BBB‐LIKE ENGINEERING

Vascularization is not only a strategy for improving organoid survival; it is also a translational bottleneck because it affects neurovascular signaling, BBB maturation, immune–cell interactions, and drug delivery.^8^, ^55^ Therefore, vascularized organoids should be evaluated using both structural and functional readouts.^55^, ^58^

Although current brain organoids recapitulate many key events of human brain development, they lack the in vivo microenvironment and a functional, perfusable vasculature. Under standard culture conditions, cerebral organoids can be maintained in vitro for up to 1 year; however, growth typically plateaus after several months, and necrosis arises within the core.^43^, ^59^ Physiologically, the brain is a highly energy‐demanding organ with rapid metabolic turnover and therefore requires a dense capillary network to supply oxygen and nutrients. As a result, avascular brain organoids experience nutrient and oxygen limitations, leading to restricted cell proliferation, reduced viability in interior regions, premature differentiation, impaired neurogenesis, and aberrant cortical development.^60^ In humans, brain development and vascularization proceed concurrently, and the absence of a vascular system can result in malformations. Consistent with this, studies of the developing rodent brain indicate that blood vessels not only provide metabolic support but also regulate neuronal differentiation, migration, and circuit assembly.^61^, ^62^ Furthermore, the vascularization of brain organoids helps preserve their cellular architecture and promotes the formation of a functional neurovascular unit and the blood–brain barrier. Collectively, these observations underscore the central importance of vascularization for brain development and organoid maturation.

Vascularization of human brain organoids is achieved via two principal strategies: creating a vascular network in vitro or inducing vascularization in vivo following transplantation. For in vivo vascularization, fetal or adult tissues were grafted into the brains of rodents in early work.^63^ In such transplants, the host vasculature efficiently infiltrates the implanted brain organoids; vascular ingrowth is typically detectable within 7–10 days post‐implantation, and extensive vessel formation becomes evident by day 14. Two‐photon microscopy reveals robust blood flow within the infiltrating vessels.^64^, ^65^ Greater degrees of vascularization correlate with greater engraftment success. For example, when human brain organoids were transplanted into the posterior cortex of NOD‐SCID mice, the host vessels penetrated the grafts within 14 days, and the organoids survived for up to 233 days. Notably, 85.4% of the grafts became vascularized, whereas nonvascularized organoids failed to survive. Compared with age‐matched, nonvascularized organoids maintained in vitro, vascularized grafts presented reduced apoptosis, larger sizes, and significantly greater numbers of mature neurons.^65^, ^66^ Vascularization strategies introduce vascular lineage cells or mesodermal progenitor‐derived vascular components into brain organoids in vitro.^56^ Embedding organoids in Matrigel containing hiPSC‐derived endothelial cells promotes intratissue vessel growth.^67^ The induction of endothelial transcription factors, such as ETV2, in stem‐cell‐derived organoids has been reported to promote vascular‐like network formation, improve tissue survival, and support neuronal maturation. The induced endothelial‐like cells express tight‐junction and transporter‐related markers and show several BBB‐associated structural features, but these findings should be interpreted as BBB‐like properties rather than complete BBB reconstruction.^8^ Another approach combines brain organoids with vascular spheroids to generate vascularized constructs; upregulation of the Wnt/β‐catenin pathway further promotes vascular formation within organoids.^8^, ^68^

Increasing organoid vascularization enables more reliable in vitro models for investigating human central nervous system development, disease progression, and translational applications.^8^

For BBB‐like models, functional interpretation requires integration of endothelial identity, barrier‐associated markers, and functional assays. Detailed marker‐based validation criteria are discussed in Section 6.3.

## 6 COMPARATIVE FUNCTIONAL VALIDATION FRAMEWORK FOR BRAIN ORGANOIDS

Functional validation should be matched to the biological claim being made. Brain organoids cannot be judged by morphology alone, and no single readout is sufficient to define maturity or translational reliability. A rigorous validation framework should integrate morphology, lineage identity, tissue health, synaptic organization, electrophysiological activity, neurochemical signaling, BBB‐like function, and molecular benchmarking.

### 6.1 Morphology, growth, and quality control

The formation of brain organoids is a gradual process. Currently, the three‐dimensional structure of brain organoids allows significant tissue architecture, but the culture of these organoids is often hindered by a lack of sufficient oxygen and nutrients.^69^ There is a cluster of specific cells, such as radial glial cells, that can survive in deeper regions of the organoid, likely due to their distinct metabolic demands and elongated shape, which allows them to interact with the external environment differently. However, as the number of neurons increases, they are gradually compressed toward the interior, leading to necrosis due to insufficient nutrients. As a result, a slicing culture technique has been developed, which exposes the internal part of the organoid to air and nutrients, potentially improving cell survival rates.^59^ This slicing culture method also allows for morphological analysis of the success of brain organoid construction.

Morphological assessment should instead focus on brain organoid‐specific quality‐control endpoints, including organoid size, projected area, volume, surface regularity, neuroepithelial bud formation, ventricular zone‐like structures, regional thickness, and necrotic or cystic regions.^16^, ^70^ These measurements are useful for evaluating growth kinetics and batch consistency, but they cannot establish regional identity, neuronal maturity, synaptic function, or BBB‐like activity.^70^ Molecular stress states and impaired subtype specification should also be considered when interpreting organoid quality and maturation.^60^ Table 1 summarizes these morphology‐based quality‐control endpoints together with complementary functional evaluation modalities.

**TABLE 1:** Structural and functional quality‐control framework for brain organoid validation.

Validation domain	Main measurements	What they evaluate	Key limitation
Whole‐organoid growth	Organoid size, projected area, perimeter, volume	Growth kinetics and gross developmental progression	Size alone cannot establish regional identity or functional maturation.
Shape and surface regularity	Overall shape, surface smoothness, sphericity	Gross morphology and routine batch‐quality control	Smooth or spherical morphology does not prove biological quality.
Neuroepithelial and regional organization	Neuroepithelial buds, VZ‐like structures, VZ/SVZ/CP‐like area or thickness	Early neuroepithelial organization and cortical‐like tissue patterning	These structures should be interpreted with molecular markers and spatial organization.
Tissue health	Necrotic or cystic regions, central necrosis, DAPI/Hoechst nuclear count	Tissue damage, cell density, and normalization of marker‐positive cells	Nuclear count is not an apoptosis assay; viability or apoptosis requires specific readouts.
Batch consistency	Organoid size distribution, inter‐organoid variability, marker‐positive cell proportions	Reproducibility across organoids and differentiation batches	Requires adequate organoid number, independent batches, and clear exclusion criteria.

### 6.2 Electrophysiological and electrochemical assessment of brain organoids

Electrophysiological activity is an important functional readout for evaluating brain organoids, including spontaneous baseline activity and stimulus‐evoked responses. As neuronal activity emerges, brain organoids can generate various electrophysiological signals, such as action potentials, firing activity, spike bursts, local field potentials, network bursts, and synchrony.^26^, ^71^ Quantitative parameters, including firing rate, interspike interval, spike waveform, burst frequency, local field potential dynamics, and network synchrony, provide information on neuronal excitability, synaptic connectivity,^69^ and network‐level maturation within specific brain organoid models, such as cortical, midbrain, or microglia‐containing organoids.^69^, ^72^ These measurements are useful for assessing the development of functional neuronal networks, although they should be interpreted in relation to organoid type, developmental stage, and recording method.

Currently, techniques for detecting electrophysiological activity in brain organoids include patch‐clamp recording, which enables the measurement of single‐cell electrophysiological properties in organoid‐derived neurons.^27^ This technique provides higher temporal resolution and a better signal‐to‐noise ratio (SNR). Whole‐cell patch‐clamp recording establishes a high‐resistance seal between the recording pipette and the cell membrane, allowing membrane potential or ionic currents to be measured under current‐clamp or voltage‐clamp configurations, respectively.^73^ Xu et al. used whole‐cell patch‐clamp to investigate the presence of functionally mature neurons in microglia‐containing organoids. In three‐dimensional microglial organoids, neurons exhibit functional maturation, displaying voltage‐dependent K^+^ and Na^+^ currents, generating spontaneous action potentials, and producing repetitive evoked action potentials in response to step current injections.^72^ Similarly, Smits et al. used whole‐cell patch‐clamp to record pacemaking and evoked firing activity from TH‐positive neurons. These midbrain‐like organoids have proved useful for Parkinson's disease modeling.^74^

Microelectrode arrays provide non‐invasive, longitudinal recordings of extracellular spikes and local field potentials at population level.^71^, ^75^ Common measures include firing rate, burst frequency, network‐burst rate, active‐electrode fraction, synchrony, and evoked responses.^26^ Interpretation depends on electrode coverage, organoid geometry, coupling, detection thresholds, and developmental age; reporting several metrics is therefore more informative than relying on firing rate alone. MEA and patch clamp answer different questions: MEA follows network dynamics over time, whereas patch clamp resolves cellular excitability and synaptic events. Electrochemical or optical sensors can add information on transmitter release, including glutamate, but these measurements need electrophysiological and pharmacological controls.^76^, ^77^

### 6.3 Imaging‐based lineage and regional identity assessment

Imaging is used to quantify cell populations, marker intensity, spatial organization, migration, tissue permeability, and synaptic structures during organoid development.^78^, ^79^, ^80^ Cell and nuclear counts provide useful developmental and quality‐control information, but they should be normalized to appropriate tissue or cell‐number denominators. Two‐dimensional immunofluorescence is suitable for routine comparisons, whereas three‐dimensional and live imaging are needed when the claim concerns internal architecture, migration, or folding. The acquisition and analysis method should therefore be chosen for the biological question rather than treated as a generic measure of organoid quality.

At the level of cell types, neural stem and progenitor cells are commonly labeled with SOX2, PAX6, and Nestin, which reflect the stemness, self‐renewal capacity, and apicobasal polarity of neuroepithelial cells. Cell proliferation is usually assessed via Ki67 to mark cycling cells or phosphorylated histone H3 (pHH3) to specifically label cells in mitosis. Immature neurons are typically identified with DCX and βIII‐tubulin (TUJ1), whereas mature neurons are labeled with MAP2 or NeuN; these markers are associated with microtubule stabilization in axons and dendrites, increased neurite complexity, and terminal neuronal differentiation.^2^, ^4^, ^81^ Astrocytes are commonly detected by GFAP, S100β, and ALDH1L1, which together indicate the generation and maturation of astrocytes as well as their roles in metabolic support and synaptic modulation.^82^ Microglia are usually identified with IBA1, TMEM119, P2RY12, and CD68, a combination that distinguishes homeostatic from activated states and reflects surveillance, inflammatory responses, and phagocytic activity.^83^, ^84^ Neuroepithelial polarity can be assessed using apical junctional markers such as N‐cadherin and ZO‐1 in ventricular‐like structures.^2^, ^16^ In BBB‐like organoid systems, endothelial identity and barrier‐associated features should be evaluated using CD31, VE‐cadherin, ZO‐1, Claudin‐5, GLUT1, P‐gp, permeability assays, and TEER measurements.^8^, ^58^, ^85^ Comparing the proportion and spatial distribution of these marker‐positive cells at different time points allows quantitative assessment of key developmental events, including neurogenesis, gliogenesis, microglial colonization, and barrier formation. Single‐cell transcriptomic and chromatin‐accessibility analyses can further benchmark organoid cell states against developing human brain references.^86^ The marker‐based validation readouts discussed above are summarized in Table 2.

**TABLE 2:** Marker‐based validation readouts for evaluating brain organoid lineage identity, regional organization, and BBB‐like phenotypes.

Validation domain	Markers mentioned in this review	What they support	Key limitation
Neural stem/progenitor and proliferation	SOX2, PAX6, Nestin, Ki67, pHH3	Neural progenitor identity, VZ‐like organization, and proliferative activity	These markers do not prove regional identity or maturation by themselves.
Neuronal differentiation and maturation	DCX, TUJ1, MAP2, NeuN	Early neurogenesis, neuronal differentiation, and supportive evidence of neuronal maturation	Marker expression does not demonstrate functional synaptic or electrophysiological maturity.
Glial and immune components	GFAP, S100β, ALDH1L1, IBA1, TMEM119, P2RY12, CD68	Astrocyte generation, microglial incorporation, and activation‐related phenotypes	Marker expression should be interpreted with developmental stage, morphology, and functional assays.
Neuroepithelial polarity	N‐cadherin, ZO‐1	Apical junctional organization and ventricular‐like lumen formation	These markers support epithelial polarity but should not be interpreted as BBB function.
Endothelial and BBB‐like phenotype	CD31, VE‐cadherin, ZO‐1, Claudin‐5, GLUT1, P‐gp	Endothelial identity, junctional organization, and BBB‐associated phenotypic features	Marker expression alone is insufficient; permeability, TEER, transporter activity, or drug‐transport assays are required.

Intensity quantification involves analyzing the molecular components of cells in the organoid culture and their proportions, allowing for evaluation of organoid development by measuring the relative amounts of different markers.^78^ Morphological analysis typically involves measuring 2D parameters (such as length, perimeter, and area) and 3D parameters (such as volume and sphericity) to observe the overall growth of the brain organoid. Confocal microscopy is used to measure the perimeter of the culture,^79^ which helps assess the shape parameters of early brain organoids. Additionally, measuring the surface area better highlights the shape characteristics of the organoids, especially their nonspherical shapes, enabling comparisons of growth differences under various environmental conditions. At the sample level, 3D reconstruction of the surface area from light‐sheet microscopy images is possible,^80^, ^87^ or the area of sections can be measured to track the growth of specific regions (e.g., the VZ, SVZ, and CP).^88^

Currently, the primary equipment used for obtaining brain organoid images is a confocal microscope, which can display fluorescence images of immunomarker expression. However, confocal microscopy is limited by its use of lower‐wavelength excitation light (typically 405–650 nm), which is more readily scattered and absorbed in biological tissue, thus resulting in relatively shallow penetration depths. Therefore, in recent years, scientists have turned to multiphoton microscopy for imaging. A significant advantage of multiphoton microscopy is its use of longer wavelength excitation light, which not only reduces scattering in biological samples but also typically allows for deeper imaging. Additionally, multiphoton microscopy offers superior depth resolution, enabling high‐quality imaging much deeper within tissue. This capability also allows for the acquisition of unique “side‐view” images of the sample.^78^, ^79^, ^80^, ^89^ For long‐term, real‐time imaging, advanced imaging techniques and optical clearing technologies must be combined to develop imaging platforms for brain organoid studies. For example, Karzbrun et al. encapsulated brain organoids in microfabricated chambers on a chip with a height of 150 μm and imaged them over several weeks to explore the physical properties of brain folding.^90^ This technique enables long‐term in situ imaging. These imaging technologies allow for more in‐depth observation and analysis of the microstructure and dynamic cellular organization of brain organoids under in vitro culture conditions, providing useful tools for dynamic structural and cellular analysis of brain organoids.

## 7 BIOMEDICAL AND TRANSLATIONAL APPLICATIONS

In recent decades, with the gradual maturation of brain organoid technology, these organoids have demonstrated broad potential in various research disciplines and clinical applications. Their advantages lie in their ability to mimic physiological tissue structures and partially replicate brain function and activity.^91^ We summarize the applications of brain organoids in brain development, neurological disease modeling, drug screening, and artificial intelligence.

The evidentiary requirements differ across applications: developmental and disease models require genotype–phenotype concordance and appropriate controls; drug screens require reproducible quantitative endpoints, exposure control, and orthogonal confirmation; and biohybrid systems require stable stimulation‐recording interfaces and computational baselines. The following examples are therefore interpreted according to their intended use rather than as equivalent demonstrations of organoid maturity.

### 7.1 Developmental biology and genetic disease modeling

Brain organoids have opened new avenues for studying brain development (Figure 6a). While the tumor suppressor gene p53 (TP53) has been widely studied in cancer progression and tumorigenesis, its specific function in human brain development remains poorly understood. Recently, Navarro et al. used brain organoids to explore the role of TP53 in human brain development.^92^ Researchers have transduced control short hairpin RNA (shCtrl) and TP53‐targeted short hairpin RNA (shTP53) into human induced pluripotent stem cells, generating control and TP53 knockdown (TP53KD) brain organoids. After 30 days of culture, immunohistochemical analysis revealed that the neural stem cell (NSC) layer in the TP53KD organoids was disorganized, with SOX2‐positive NSCs distributed in both the inner and outer regions of the renal tubu‐like area. Furthermore, compared with control organoids, TP53KD organoids presented significant reductions in TBR1, postmitotic neuron, TBR2, and intermediate progenitor cell numbers. Navarro et al. did not observe significant differences in NSC proliferation or apoptosis between the two groups. To further investigate whether TP53 regulates the cell cycle status in organoids, researchers have analyzed the cell cycle distribution. They reported an accumulation of cells in the G1 phase and a corresponding reduction in the S phase in TP53KD organoids. This change suggests that TP53 deletion may disrupt normal cell cycle progression, indicating its crucial role in regulating the cell cycle during brain development. This case illustrates how brain organoids can aid in the study of genetic and molecular issues through the use of cellular or animal models. In addition to TP53, brain organoids have been widely used to study the function of genes involved in the regulation of brain size and microcephaly during development. Lancaster et al. first established a human pluripotent stem cell‐derived brain organoid system and utilized patient‐derived iPSCs carrying CDK5RAP2 mutations to construct a microcephaly organoid model. They reported that the mutant organoids presented a significantly reduced overall volume and a thinner neuroepithelial region, providing a mechanistic basis for the disease phenotype. This case demonstrates the unique value of brain organoids over traditional animal models in elucidating the molecular mechanisms of genetic diseases.^2^

### 7.2 Neurological disease modeling

Neurodegenerative diseases (NDDs) are a group of disorders characterized by the progressive loss of neurons or myelin, primarily manifesting as cognitive or motor dysfunction. Alzheimer's disease (AD) is a typical NDD characterized by a gradual decline in cognitive function, behavioral abnormalities, and persistent deterioration of physical ability.^93^, ^94^

Familial Alzheimer's disease (FAD) has been closely linked to mutations in the amyloid precursor protein (APP) gene and presenilin 1 (PS1) gene.^94^, ^95^ Three‐dimensional human neural culture systems have been used to model AD‐related Aβ deposition and tau pathology, whereas human cerebral organoids have further been developed to model amyloid‐β‐ and tau‐associated pathological changes in a more tissue‐like context.^96^ These models provide useful in vitro platforms for studying AD‐related pathological features and neurodegenerative disease‐associated phenotypes in a tissue‐like context (Figure 6c).^93^ Furthermore, researchers generated brain organoids from iPSCs derived from familial AD patients and observed pathological abnormalities such as amyloid plaque deposition and neurofibrillary tangles in these organoids.^96^ The use of 3D brain organoids derived from patient somatic cells provides a feasible platform for discovering target drugs and effective therapeutic interventions.

Parkinson's disease is another common NDD characterized by reduced dopamine levels in the substantia nigra and disrupted fine motor control functions within the basal ganglia, leading to clinical symptoms such as bradykinesia, muscle rigidity, and resting tremors.^97^ Recently, Kim et al. generated 3D midbrain organoids derived from iPSCs with the LRRK2 G2019S mutation to understand how the LRRK2 gene mutation leads to Parkinson's disease. This team employed a novel approach in which iPSCs derived from Parkinson's disease patients carrying the G2019S mutation were used to create 3D midbrain organoid models.^98^ The study revealed that by day 60 of organoid development, dopaminergic neuron function within these organoids was significantly impaired. This was evident by a marked decrease in the expression levels of key markers such as TH, AADC, and DAT compared with those in control organoids without the mutation. Among these enzymes, tyrosine hydroxylase (TH) is the rate‐limiting enzyme in catecholamine biosynthesis, whereas aromatic L‐amino acid decarboxylase (AADC) catalyzes the conversion of L‐DOPA to dopamine. Together, TH and AADC are commonly used to assess dopaminergic lineage differentiation and dopamine‐synthesis capacity.^99^ Vesicular monoamine transporter 2 (VMAT2) is located on the synaptic vesicle membrane, where it actively transports and sequesters newly synthesized dopamine into the vesicle. In contrast, the dopamine transporter (DAT) resides on the presynaptic terminal plasma membrane and is responsible for the rapid reuptake of dopamine from the synaptic cleft. Together, these two proteins reflect the integrity of the vesicular storage and presynaptic reuptake machinery.^100^ Furthermore, these organoids recapitulated typical Parkinson's disease pathology, showing abnormal accumulation of phosphorylated α‐synuclein at the S129 site, along with increased mitochondrial autophagy activity, as evidenced by the expression of related autophagy markers. A key finding was that treatment with an LRRK2 kinase inhibitor effectively reduced the accumulation of phosphorylated α‐synuclein and alleviated neuronal damage. These results indicate that G2019S‐LRRK2 mutant midbrain organoids reproduce selected Parkinson's disease‐related phenotypes and may provide a platform for mechanistic studies and therapeutic evaluation.^91^, ^98^

### 7.3 Drug screening and precision medicine

In recent years, with the maturation of brain organoid research, they have played a significant role in drug screening. For example, 3D brain organoids derived from PSCs have been used for application‐specific pharmacological testing and disease‐related drug screening (Figure 6b).

Brain organoid‐based drug screening is most convincing when the screening strategy is linked to a defined disease phenotype and matched functional readouts. For example, brain‐region‐specific and self‐organized cerebral organoids have been used to model ZIKV‐associated neurodevelopmental injury and to evaluate candidate therapeutic responses.^4^, ^88^ Patient‐derived glioblastoma‐cerebral organoid co‐culture models can preserve aspects of tumor invasion and tumor–brain interaction, providing a more relevant platform for evaluating anticancer drug responses than conventional two‐dimensional cultures.^101^ For compounds requiring CNS delivery, BBB‐on‐chip or BBB‐like organoid systems may provide complementary permeability and barrier‐function readouts, but these platforms should be clearly distinguished from brain organoid models themselves.^8^, ^58^

### 7.4 Exploratory biohybrid systems and organoid–computer interfaces

Brain organoids have recently been explored in artificial intelligence‐related research through organoid‐based biological computing and hybrid bioelectronic interface systems (Figure 6d).^102^ In these platforms, brain organoids are coupled with multielectrode arrays or related recording interfaces, allowing electrical stimulation to be delivered as input and organoid‐derived electrophysiological responses to be recorded as output. The resulting spatiotemporal activity patterns can then be analyzed by computational readout algorithms for tasks such as signal classification, pattern recognition, and time‐series prediction. This framework provides a possible route for linking living neuronal networks with machine‐based computation, while also offering an experimental system for studying adaptive network dynamics in vitro.

**FIGURE 5:**
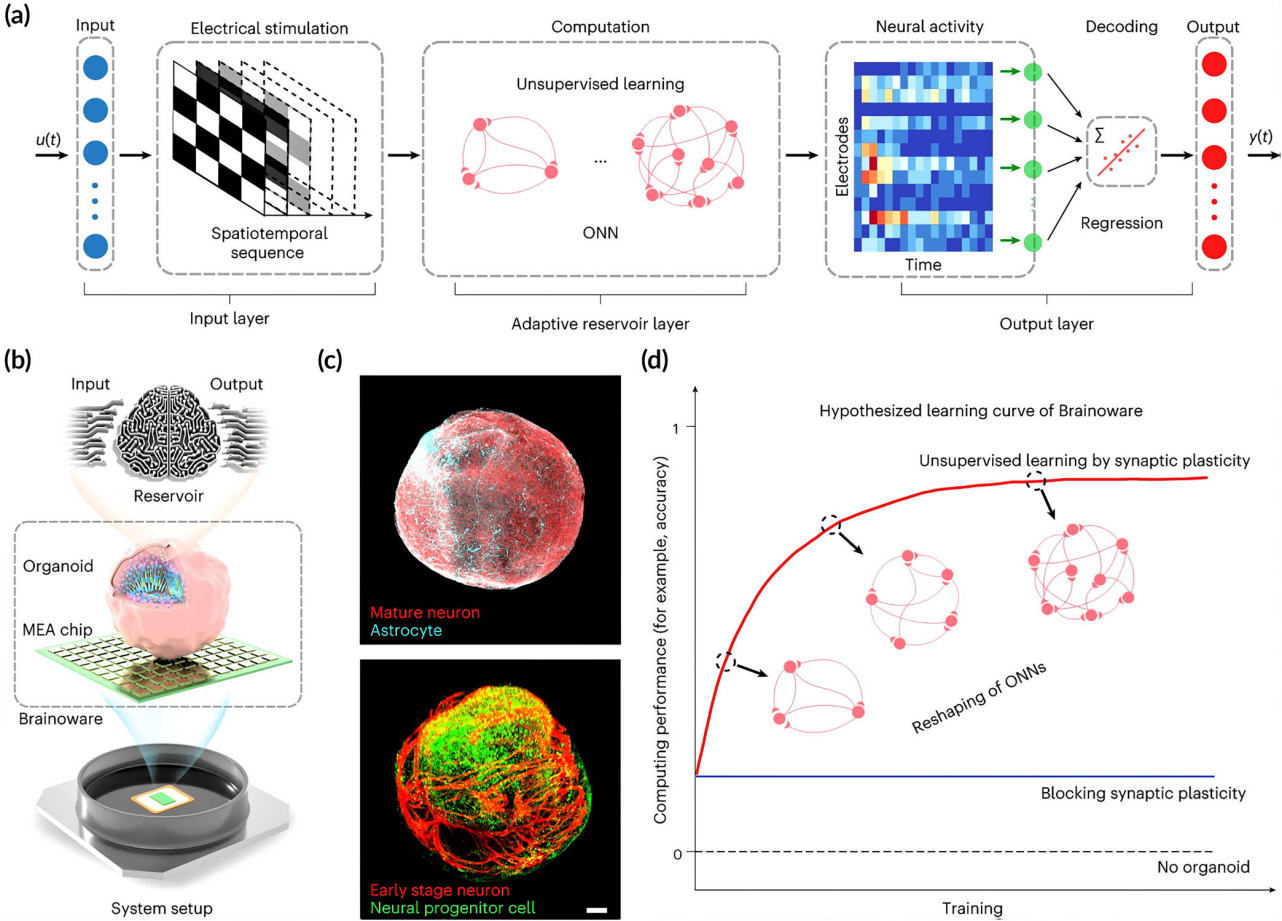
Brainoware‐based reservoir computing using human brain organoids. (a) Schematic of the Brainoware adaptive reservoir computing framework. (b) Schematic of the Brainoware setup paradigm. (c) Cortical organoids displaying complex 3D neural networks with various brain cell types through whole‐mount immunostaining. (d) Schematic illustrating the unsupervised learning process through the remodeling of the BNN during training and the constrained state of unsupervised learning when synaptic plasticity is inhibited.^102^ Reproduced under the terms and conditions of the Creative Commons Attribution (CC BY) license.

A representative example is Brainoware, an organoid‐based reservoir computing system in which human brain organoids are integrated with high‐density multielectrode arrays (Figure 5a–d).^102^ In this system, electrical inputs are delivered to the organoid network, and the evoked electrophysiological responses are decoded by a computational readout layer. By using organoid‐derived network dynamics as a biological reservoir, Brainoware has been applied to tasks such as speech recognition and nonlinear equation prediction under defined experimental conditions.^102^ This work provides a concrete example of how brain organoids can be incorporated into AI‐related computational workflows, rather than serving only as developmental or disease models.

Organoid‐based interfaces share some technical features with conventional BCIs, particularly bidirectional stimulation and recording through MEAs.^103^, ^104^, ^105^ Their present purpose, however, is not clinical control of an external device but controlled study of activity‐dependent adaptation in vitro.

Compared with conventional BCI systems that decode neural activity from the intact or injured brain, brain organoid‐based interfaces are still mainly experimental platforms for modeling neural activity, plasticity, and network adaptation in vitro. Electrical stimulation may modulate organoid network activity and provide a controllable approach for examining activity‐dependent changes in developing neural circuits. However, its role in modeling neural repair or functional recovery should be described cautiously, because current organoid systems do not reproduce the full anatomical organization, sensory input, vascularization, immune environment, or long‐range connectivity of the human brain.^105^ Therefore, brain organoid‐BCI systems are better viewed as early‐stage biohybrid platforms for investigating stimulation–response relationships, neural decoding strategies, and adaptive network dynamics, rather than as established therapeutic systems.

Despite these advances, brain organoid‐based AI and BCI systems remain at an early stage. Current limitations include organoid‐to‐organoid variability, immature and heterogeneous network organization, limited long‐term stability of bioelectronic interfaces, and the lack of standardized stimulation, recording, and decoding protocols. In addition, ethical issues related to long‐term culture, learning‐like activity, and the interpretation of complex electrophysiological responses require careful consideration. Future studies may therefore focus on improving the reproducibility of organoid generation, developing stable bioelectronic interfaces, establishing standardized computational readouts, and defining appropriate criteria for evaluating both biological maturation and computational performance.

## 8 REPRODUCIBILITY, STANDARDIZATION, AND TRANSLATIONAL BOTTLENECKS

### 8.1 Sources of variability and minimum reporting

Reproducibility represents one of the most critical challenges limiting the scientific reliability and translational potential of brain organoid technology. Unlike conventional two‐dimensional cell culture systems with relatively defined experimental conditions, brain organoid generation is a complex developmental process involving pluripotent stem cell maintenance, embryoid body formation, neural induction, three‐dimensional self‐organization, regional patterning, and long‐term maturation. Variations introduced at any stage can accumulate and influence the final cellular composition, structural organization, maturation state, and functional properties of organoids. Therefore, variability is not merely a technical limitation but a fundamental factor determining whether findings obtained from brain organoid models can be reliably reproduced and generalized across laboratories.

The sources of variability in brain organoid systems are highly multifactorial. Biological factors, including donor genetic background, reprogramming procedures, pluripotent stem cell quality, passage number, and epigenetic memory, can affect differentiation capacity and developmental trajectories. Engineering‐related factors, including initial embryoid body size, extracellular matrix composition, hydrogel properties, Matrigel batch variation, morphogen concentration and timing, culture medium composition, oxygen and nutrient transport, and dynamic culture parameters, further influence regional identity, cell‐type composition, tissue architecture, and functional maturation. These variations contribute to substantial inter‐organoid and inter‐batch heterogeneity, making direct comparison among studies challenging.

The impact of poor reproducibility extends beyond experimental consistency. In disease modeling, uncontrolled variability may obscure genotype–phenotype relationships or generate inconsistent disease‐associated phenotypes. In drug screening, heterogeneous organoid responses may reduce screening reliability and increase the possibility of identifying model‐specific artifacts rather than biologically meaningful therapeutic effects. For BBB‐like models and neurovascular applications, differences in maturation and cellular composition may directly affect barrier properties and transport measurements. Therefore, improving reproducibility is a prerequisite for establishing brain organoids as reliable platforms for biomedical discovery.

A major step toward improving reproducibility is the establishment of comprehensive reporting standards. Current studies should provide detailed information regarding cell source and genetic background, pluripotency validation, passage number, embryoid body formation method and size distribution, extracellular matrix or hydrogel composition, differentiation protocols, morphogen dosage and timing, culture platform, oxygenation and flow conditions, organoid age, biological replicates, independent differentiation batches, exclusion criteria, imaging methods, quantitative analysis procedures, and statistical strategies. Such transparent reporting will improve cross‐study comparability, facilitate protocol optimization, and provide the foundation for future standardization frameworks.

### 8.2 Engineering solutions for standardization

While biological variation is an inherent characteristic of living systems, engineering approaches provide opportunities to reduce uncontrolled variability and establish more standardized brain organoid platforms. The future development of brain organoids requires a transition from protocol‐dependent generation toward engineering‐guided production, in which critical parameters are precisely controlled, monitored, and evaluated.

One important direction is the development of defined and reproducible microenvironments. Although Matrigel remains widely used because of its ability to support three‐dimensional neural organization, its undefined composition and batch‐to‐batch variability represent major obstacles for reproducibility and clinical translation. Synthetic and recombinant extracellular matrices with controlled mechanical properties, degradation behavior, and biochemical signals may provide more predictable environments for regulating neural differentiation and maturation. These defined materials could improve experimental consistency and facilitate the establishment of manufacturing processes suitable for large‐scale applications.

Standardization also requires improved control over the production process itself. Automated platforms for stem cell maintenance, embryoid body generation, medium exchange, and morphogen delivery can minimize operator‐dependent differences and improve batch consistency. Similarly, bioreactor, perfusion, and organoid‐on‐chip systems provide opportunities to regulate oxygen supply, nutrient transport, and metabolic waste removal, thereby improving tissue viability and maturation. However, increased engineering complexity should not be considered sufficient evidence of improvement. These systems should be validated using quantitative functional criteria, including cellular composition, electrophysiological activity, barrier properties, and molecular similarity to human developmental references. In addition, advanced quality‐control strategies based on high‐content imaging, artificial intelligence‐assisted analysis, and multi‐omics profiling may provide objective approaches for evaluating organoid quality. Automated image analysis can quantify growth dynamics, morphological changes, necrotic regions, and spatial organization, while transcriptomic and epigenomic analyses may establish molecular benchmarks for defining maturation states. Nevertheless, these computational approaches require shared reference datasets, standardized annotation criteria, and independent validation before they can be widely adopted.

Importantly, standardization should not aim to produce a single universal brain organoid model. Different applications require different biological characteristics and validation criteria. Developmental studies require accurate lineage specification; disease models require reproducible pathological phenotypes; drug screening platforms require scalable quantitative endpoints; BBB‐related systems require functional barrier assessment; and biohybrid interfaces require stable electrophysiological performance. Therefore, future standardization should focus on application‐specific acceptance criteria rather than a single definition of organoid quality.

By improving reproducibility and standardization, brain organoid technologies can become more reliable experimental systems for studying human biology. Such advances will strengthen cross‐laboratory comparability, increase confidence in disease modeling and drug discovery, support personalized medicine strategies, and accelerate the translation of organoid platforms toward practical biomedical applications.

### 8.3 Translational bottlenecks and future applications

Improving reproducibility and standardization is essential for advancing brain organoids from experimental models toward more reliable human‐relevant platforms. Nevertheless, several biological and engineering limitations continue to restrict their broader translational use. Current brain organoids remain simplified and partially immature representations of the human brain, with limitations including insufficient vascularization, incomplete BBB maturation, limited immune and stromal interactions, heterogeneous cellular composition, and restricted long‐range neural connectivity.

In disease modeling, the utility of brain organoids depends not only on their ability to reproduce disease‐associated phenotypes, but also on the stability and reproducibility of these phenotypes across different experimental conditions. Standardized generation procedures and quantitative functional assessments are therefore critical for distinguishing disease‐related alterations from variations introduced during culture and maturation. Similarly, standardized organoid platforms may complement conventional cellular and animal models in drug discovery by incorporating patient‐specific genetic backgrounds, multicellular interactions, and functional responses. However, their application in pharmaceutical development requires further optimization of drug exposure protocols, assay reproducibility, and validation against clinically relevant outcomes.

Enhancing physiological relevance remains a major objective for future brain organoid engineering. Vascularization and BBB‐related strategies, including endothelial cell incorporation, vascular‐like structure formation, perfusable microfluidic systems, and dynamic perfusion culture, have been explored to improve nutrient transport, barrier properties, and tissue maturation. Although these approaches have shown considerable potential, they remain under development, and further investigation is needed to determine how vascular components, extracellular matrix properties, mechanical signals, and perfusion conditions collectively influence neural differentiation and long‐term maturation.

**FIGURE 6:**
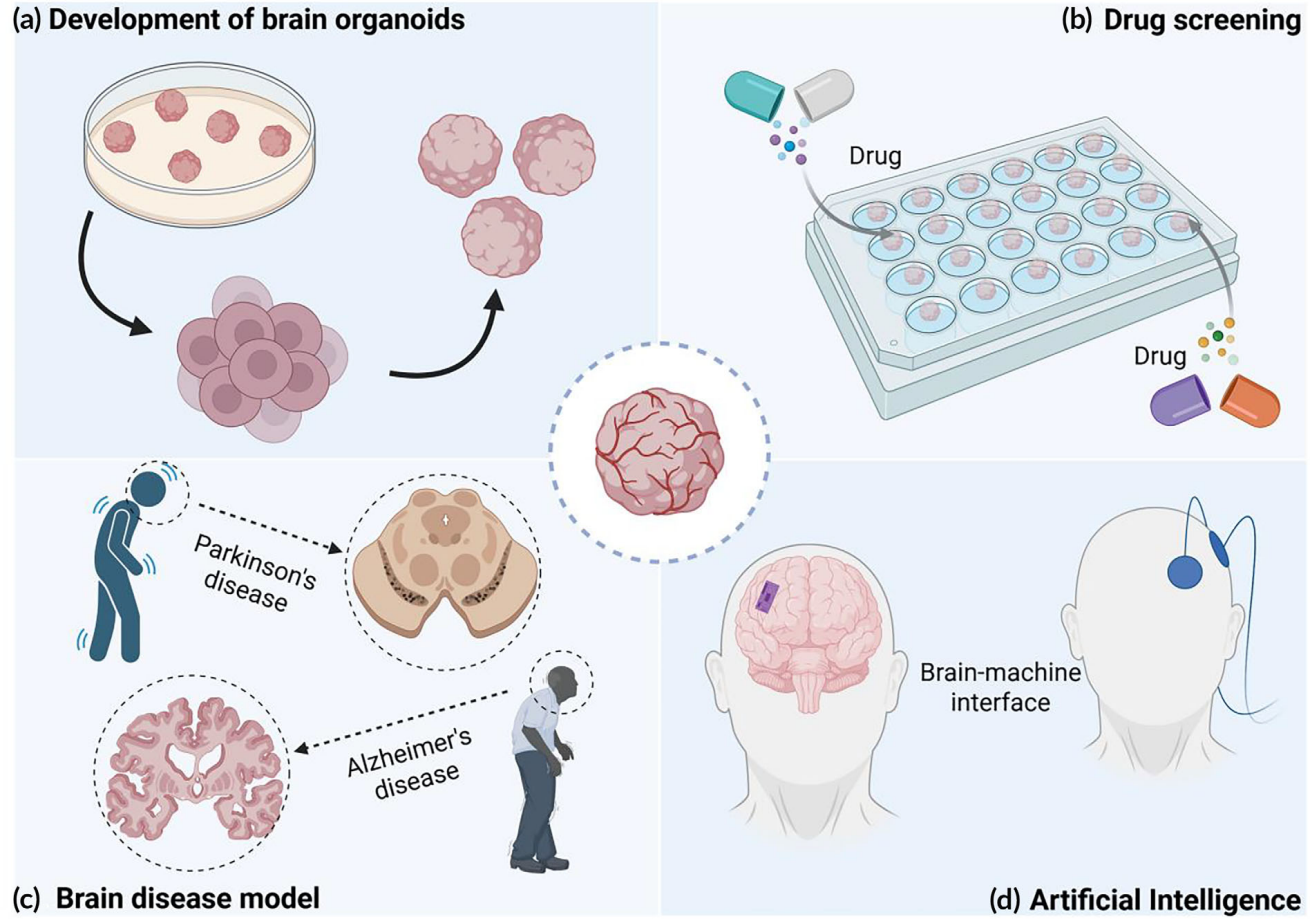
Biomedical and computational applications of brain organoids. (a) Brain organoids provide in vitro models for investigating selected aspects of human neurodevelopment, including neural progenitor expansion, neuronal differentiation, and early tissue organization. (b) Brain organoids can be applied to drug screening and pharmacological evaluation by assessing disease‐related phenotypes, treatment responses, and potential neurotoxicity. (c) Patient‐derived or genetically modified brain organoids can be used for modeling neurological diseases, including neurodevelopmental disorders, neurodegenerative diseases, and injury‐related pathological processes. (d) Integration of brain organoids with bioelectronic interfaces, multielectrode arrays, AI‐assisted analysis, and brain‐inspired computing systems provides an exploratory platform for studying neural activity patterns, adaptive network dynamics, and biohybrid computation. This figure was constructed using BioRender.

Beyond conventional biomedical applications, the integration of brain organoids with artificial intelligence, multi‐omics approaches, and bioelectronic interfaces represents an emerging direction (Figure 6). These interdisciplinary strategies may facilitate more comprehensive phenotypic characterization, cell‐type identification, electrophysiological analysis, and real‐time monitoring of organoid function. Organoid‐based computing and brain‐inspired interfaces further expand the potential applications of these models; however, their interpretation remains challenging due to immature network organization, organoid‐to‐organoid variability, limited long‐term interface stability, and ethical considerations.

For transplantation and regenerative applications, additional safety issues require careful evaluation, including residual pluripotent cells, uncontrolled growth, ectopic differentiation, immunogenicity, inappropriate circuit integration, and seizure risk. Therefore, quality assessment should be established according to application‐specific requirements rather than relying on a universal definition of organoid quality. Moving forward, the successful translation of brain organoids will depend on the integration of biological complexity with precise engineering control, enabling these platforms to provide reliable insights into human development, disease mechanisms, therapeutic responses, and emerging neurotechnological applications.

## 9 CONCLUSIONS AND OUTLOOK

Future progress will require application‐specific acceptance criteria that link engineering parameters, functional validation, and translational objectives.

Overall, brain organoids should be viewed as evolving experimental models that complement, rather than replace, animal models and conventional cell culture systems. Their future utility will depend on continued improvements in standardization, reproducibility, maturation, vascularization, functional assessment, and ethical governance. With further methodological refinement, brain organoids may contribute to more reliable in vitro models for studying human neurodevelopment, disease mechanisms, therapeutic screening, and biohybrid computational systems.

## AUTHOR CONTRIBUTIONS

**Guohong Huang:** Writing – original draft. **Chenfei Lu:** Writing – original draft. **Zihan Jin:** Writing – original draft. **Yunchuanxiang Huang:** Validation. **Xinya Du:** Methodology. **Xixi Hu:** Conceptualization. **Hui Peng:** Formal analysis. **Shanrun Wang:** Methodology. **Lin Wen:** Validation; supervision. **Juhui Qiu:** Supervision; visualization. **Guixue Wang:** Writing – review and editing; funding acquisition; investigation. **Chuanrong Zhao:** Writing – review and editing; funding acquisition; investigation.

## CONFLICT OF INTEREST STATEMENT

The authors declare that they have no conflicts of interest.

## Data Availability

The data that support the findings of this study are available from the corresponding author upon reasonable request.
